# Activation of the Alternative NFκB Pathway Improves Disease Symptoms in a Model of Sjogren's Syndrome

**DOI:** 10.1371/journal.pone.0028727

**Published:** 2011-12-09

**Authors:** Adi Gilboa-Geffen, Yochai Wolf, Geula Hanin, Naomi Melamed-Book, Marjorie Pick, Estelle R. Bennett, David S. Greenberg, Susan Lester, Maureen Rischmueller, Hermona Soreq

**Affiliations:** 1 Department of Biological Chemistry, The Hebrew University of Jerusalem, Jerusalem, Israel; 2 Rheumatology Unit, Queen Elizabeth Hospital, Woodville, Australia; 3 The Edmond and Lily Safra Center of Brain Sciences, The Hebrew University of Jerusalem, Jerusalem, Israel; University of London, St George's, United Kingdom

## Abstract

The purpose of our study was to understand if Toll-like receptor 9 (TLR9) activation could contribute to the control of inflammation in Sjogren's syndrome. To this end, we manipulated TLR9 signaling in non-obese diabetic (NOD) and TLR9^−/−^ mice using agonistic CpG oligonucleotide aptamers, TLR9 inhibitors, and the in-house oligonucleotide BL-7040. We then measured salivation, inflammatory response markers, and expression of proteins downstream to NF-κB activation pathways. Finally, we labeled proteins of interest in salivary gland biopsies from Sjogren's syndrome patients, compared to Sicca syndrome controls. We show that in NOD mice BL-7040 activates TLR9 to induce an alternative NF-κB activation mode resulting in increased salivation, elevated anti-inflammatory response in salivary glands, and reduced peripheral AChE activity. These effects were more prominent and also suppressible by TLR9 inhibitors in NOD mice, but TLR9^−/−^ mice were resistant to the salivation-promoting effects of CpG oligonucleotides and BL-7040. Last, salivary glands from Sjogren's disease patients showed increased inflammatory and decreased anti-inflammatory biomarkers, in addition to decreased levels of alternative NF-κB pathway proteins. In summary, we have demonstrated that activation of TLR9 by BL-7040 leads to non-canonical activation of NF-κB, promoting salivary functioning and down-regulating inflammation. We propose that BL-7040 could be beneficial in treating Sjogren's syndrome and may be applicable to additional autoimmune syndromes.

## Introduction

Sjögren's syndrome (SjS) is a common syndrome affecting various exocrine cells. It emerges at all ages, peaking in the fourth and fifth decade, and has a female to male ratio of 9∶1 [1]. SjS is characterized by extraglandular manifestations, severe salivary gland hypo-function, infiltration of lymphocytes and plasmacytoid dendritic cells (pDCs) and decreased salivary secretion [2]. Diverse auto-antibodies indicating B cells activation and loss of immune tolerance are characteristic of SjS [3]. Serum auto-antibodies directed to the ribo-nuclear proteins SS-A/Ro and SS-B/La are believed to affect disease pathogenesis via Toll-like receptor (TLR) signaling [4].

TLRs expressed on the cell surface or endosomal membranes of innate immune system cells detect pathogens and inherent apoptotic processes releasing DNA fragments [5]. Activation of these receptors can lead to interferon (IFN)-mediated stimulation of adaptive immunity, pro-inflammatory reactions mediated by the transcriptional regulator NFκB, and possibly an additional, alternative anti-inflammatory activation of NFκB [6], [7].

Of the different TLRs, TLR9 and TLR7 are considered most relevant to autoimmunity [5], [7]. TLR9 discriminates un-methylated CpG dinucleotides, common in the genomes of most bacteria and DNA viruses, from the methylated ones in mammalian DNA [8]. Viral CpG DNA as well as apoptotic host DNA sequences can rapidly activate TLR9 in pDCs. In SjS, pDCs infiltrate the salivary gland, inducing high levels of IFNα. This promotes cellular maturation and survival of B cells, which produce auto-antibodies to the apoptotic material, worsening disease symptoms and decreasing saliva secretion [9]. TLR9-driven pro-inflammatory activation of NFκB may link between the innate and adaptive immune systems by activating T and B lymphocytes through their antigen and co-stimulatory receptors. However, NFκB also activates an alternative or homeostatic pathway which leads to suppression of pro-inflammatory cytokine production 7,10.

In response to inflammatory stimuli, pDCs express the enzyme indoleamine 2,3-dioxygenase (IDO), which mediates conversion of the essential amino acid tryptophan to kynurenine. Modulation of tryptophan catabolism by IDO is a general mechanism of action of regulatory T cells (Tregs) via activation of FOXp3. Synthetic tryptophan metabolites have correspondingly been successful in treating autoimmune neuroinflammation, apparently caused by the induction of Treg activity [10], [11]. Therefore, we predicted that the first two modes of TLR9 activation accelerate disease symptoms, whereas blocking these pathways, or activating the putative alternative NFκB pathway, may improve salivation.

ACh release from the vagus initiates and monitors saliva secretion by binding to and activating the muscarinic acetylcholine (ACh) receptor M3R [3], which stimulates intracellular release of Ca^++^ stores, activates K^+^ and Cl^−^ channels and enhances fluid secretion [12]. Many SjS patients express auto-antibodies against M3R which could inhibit parasympathetic neurotransmission to secreting epithelia [13]. Correspondingly, salivary gland cells from SjS patients require a 10-fold greater ACh concentration than control cells to elicit the same increase in Ca^++^ [14]. This may explain why there is no meaningful correlation between the degree of glandular destruction seen in biopsies and impaired salivation in SjS [2].

ACh has also been shown to activate the α7 nicotinic ACh receptor (nAChRα7) in lymphocytes and monocytes [15] leading to down-regulation of NFκB signaling and a decrease in secretion of pro-inflammatory cytokines [16], [17].

Several discriminative subtypes of TLR9 activators can activate human pDCs. Type-A CpG oligodeoxynucleotides (ODNs) often spontaneously form large multimeric aggregates and are therefore retained in the early endosomes of pDCs for relatively long periods, leading to extended activation of the signal-transducing complex and robust type α interferone (IFNα) production [18]. In contrast, Type-B CpG ODNs remain monomeric and are rapidly trafficked from early to late endosomes, making them poor producers of IFNα but strong stimulators of B cells [18]. Other ODNs assume mixed properties, among them BL-7040, previously known as hEN101, an antisense oligonucleotide against AChE mRNA which can potentiate cholinergic signaling [19].

In the current study we surmised that functional attenuation of autonomic neurotransmission to salivary glands, together with infiltration of pDCs to the gland, contribute to the onset and severity of the disease. More specifically, we predicted that SjS involves TLR9 failure, which together with ACh depletion and acetylcholinesterase (AChE) excess [16] could reduce saliva secretion. We thus studied the molecular mechanisms underlying the effect of TLR9 activation on salivary functioning by using ODN activators and blockers of TLR9 [20] in cultured cells and mouse models with modified immune functions [21].

## Results

### BL-7040 is a TLR9 agonist

**Figure 1:**
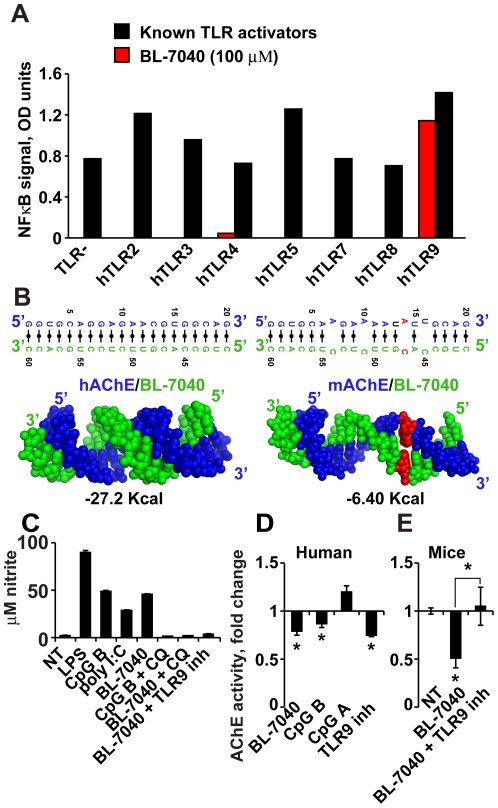
BL-7040 is a TLR9 agonist. A. Luciferase reporter quantification of NFkB activation in HEK-293 cell lines stably expressing specific TLR proteins and treated with the established agonists and BL-7040. TLR activation is given as optical density values (OD). For the HEK293-TLR2,3,4,5,7,8 and 9 the known ligands were PAM2, Poly I:C, LPS, Flagellin, R848, and a Type-B CpG (ODN 1826), respectively. HEK-293 cells expressing the reporter gene alone (TLR-) were used as a negative control. B. Molecular modeling [22] of BL-7040 (green) interaction with human AChE mRNA (blue; −27.2 Kcal) compared to mouse (m)AChE (−6.46 Kcal). The A/C mismatch is shown in red. C. RAW 264.7 murine macrophage-derived cells endogenously expressing TLR 3, 4 and 9 were treated with BL-7040 (1 µM), LPS (1 µg/ml), CpG-B (1 µM), poly I:C (2.5 µg/ml) and nitrite levels were determined. The effect of BL-7040 was abolished by co-administration of the endosomal maturation inhibitor chloroquine (CQ; 10 µg/ml) (p = 0.05) and the TLR9 inhibitor (ODN2088, 0.5 µM) (p = 0.05). Error bars represent mean±SEM. D. CpG-B (p = 0.04), BL-7040 (p = 0.05) and the human TLR9 inhibitor (p = 0.04) but not the CpG-A decreased AChE activity in human mononuclear cells. Error bars are mean±SEM. P-values (Student's t-test) marked by stars above specific columns show differences from control levels, whereas p-values marked above line bridges between columns refer to differences between the groups presented by those columns. E. Mononuclear cells from C57BL mice displayed decreased AChE activity when treated with BL-7040 (p = 0.02), but this decline was preventable when cells were co-treated with a TLR9 inhibitor (p = 0.005). Bars are mean±SEM.

The BL-7040 oligonucleotide targets human AChE mRNA. It was designed as an antisense molecule, and correspondingly reduces AChE activity in various primate systems [19], [22]. In *myasthenia gravis* patients with characteristic muscle fatigue, orally-delivered BL-7040 (0.1 µM) improves muscle tone within 30–60 minutes and for 24 hours at least [22]. The rapid action, low dose and long-lasting effect raised our concern that additional non-antisense properties of BL-7040 are involved. To test for possible TLR-mediated attributes, we administered BL-7040 (100 µM) to HEK-293 cell lines, each stably expressing a given TLR protein (TLR 2, 3, 4, 5, 7, 8 or 9) as well as a luciferase reporter plasmid under an NFκB promoter. In addition, we applied the appropriate positive ligand control to cell lines expressing the various TLRs, as described in the methods section (PAM2, Poly I:C, LPS, Flagellin, R848, and ODN 1826 for TLR 2,3,4,5,7,8 and 9 respectively). As can be seen in Fig. 1A, each ligand selectively activates its TLR target. Furthermore, only the cell line expressing TLR9 but no other TLR was activated by BL-7040, (indicating that BL-7040 is a specific TLR9 activator, and ruling out the possibility of other TLR involvement.

Within its 20-nucleotide long sequence, the human-targeted BL-7040 carries 4 mismatches compared to the mouse AChE mRNA (Fig. 1B). This would weaken its interaction with murine AChE mRNA, minimizing but not excluding the possibility that BL-7040 could have an antisense effect in mice (as indicated by molecular modeling, Fig. 1B). Addition of BL-7040 (1 µM) to cultured RAW 264.7 cells which display TLR 3, 4 and 9 [23] induced nitric oxide (NO) production similar to LPS (1 µg/ml), CpG-B (1 µM), and poly I:C (2.5 µg/ml). Furthermore, BL-7040-inducible NO production was abolished by the endosome maturation inhibitor Chloroquine (CQ; 10 µg/ml) and the TLR9 oligonucleotide inhibitor ODN2088 (molar ratio 2∶1) (Fig. 1C), demonstrating that this response is due to the TLR9 activating features of BL-7040.

Primary mononuclear cells from human blood (peripheral blood mononuclear cells, PBMC) treated with BL-7040, Type-B CpG or the human TLR9 inhibitor showed decreased AChE activity, in contrast to a non-significant increase which was seen after treatment with Type-A CpG. (Fig. 1D), reinforcing the interrelationship between TLR9 activation and the cholinergic pathway. Similarly, treatment of PBMC from C57BL mice with BL-7040 decreased AChE activity. Co-treatment with BL-7040 and a TLR9 inhibitor (ODN2088) prevented this decrease in AChE (Fig. 1E), suggesting again that BL-7040 exerts this effect through the TLR9 pathway. In mononuclear cells CpG-A activates the IRF7 pathway, triggering an increase in IFNα levels and elevating AChE activity, whereas CpG-B activates the classical NFκB pathway and triggers secretion of proinflammatory cytokines [5]. The observed reduced AChE levels called for an alternative explanation.

Searching for a possible structural basis for these intriguing properties of BL-7040, we subjected the BL-7040 oligonucleotide to heat denaturation, which abolished roughly half of NO production in BL-7040-treated RAW cells (Figure S1A). In addition, mass spectrometry analysis demonstrated that BL-7040 includes heavier than monomeric forms which were largely dissipated following the heating, suggesting that BL-7040 multimers are responsible for the TLR9 activation effect (Figure S1B). Furthermore, induction of NO by BL-7040 was sensitive to both chemical and sequence modifications. In RAW cells, BL-7040 with a phosphorothioate backbone of the three 3′ bases induced less NO production than BL-7040 with three 3′ 2-O-methyl bases. Also, changing the terminal ends of BL-7040 (bases 1–4 and 19–20) while keeping the 2-O-methyl protection was ineffective with regards to NO production, but mutating the central bases (5–15) while keeping the 2-O-methyl protection substantially reduced NO production (Figure S1C). Together, these findings indicate that the TLR9-activating capacity of BL-7040 involves heat-sensitive multimeric forms and the 10 central nucleotides of this 20-mer molecule.

### BL-7040 induces saliva secretion

**Figure 2:**
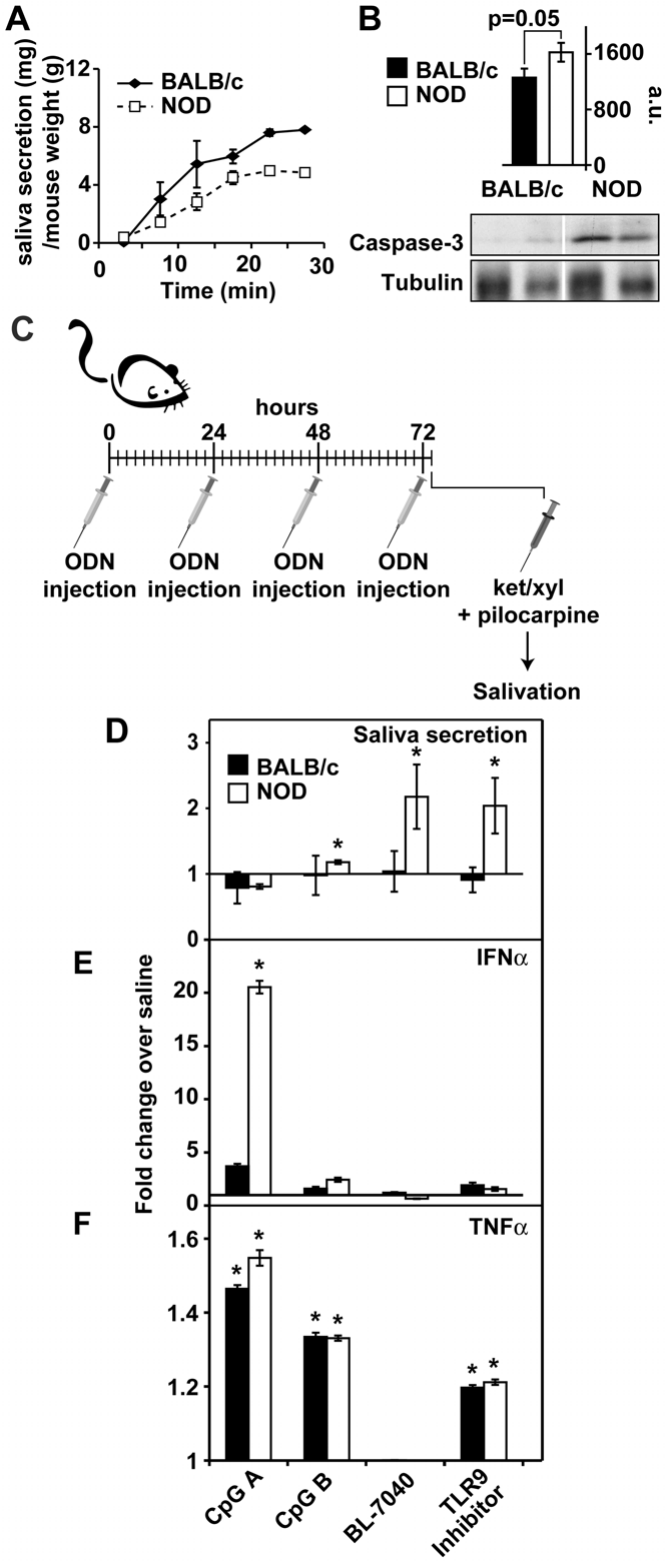
BL-7040 induces saliva secretion and reduces inflammatory markers. A. NOD mice show reduced salivary response to pilocarpine compared to BALB/c controls (p = 0.05). P-values were generated by Student's t-test. B. Western blot of salivary gland extracts from NOD mice show elevated cleaved caspase-3 levels compared to BALB/c controls, suggesting increased TLR9 activation, possibly by apoptotic DNA (p = 0.05). C. Experimental layout: NOD and the parent strain BALB/c mice were injected intraperitoneally with the noted ODNs for 4 consecutive days. Two hours after the last injection mice were anesthetized with ketamine/xylazine (Ket/xyl) and injected with pilocarpine. Saliva secretion and cytokine levels in salivary gland extracts were determined as detailed in the Methods section. D. Measurements of saliva secretion in the injected mice. NOD mice treated with the TLR9 agonist CpG-A displayed decreased saliva secretion compared to BALB/c controls (p = 0.02). CpG-B increased saliva secretion in NOD (p = 0.003) as did BL-7040 (p = 0.05) and and TLR9 inhibitor (p = 0.0018) compared to BALB/c (p = 0.05) mice. Bars are mean±SEM. E. F. IFNα and TNFα levels in injected mice. CpG-A increased both IFNα (p = 0.0001) and TNFα (p = 0.04) and levels in NOD mice. IFNα levels were also increased in BALB/C (p = 1.5*10-7) mice. TLR9 inhibitor increased TNFα levels (p = 0.005) while maintaining unchanged IFNα in both NOD and BALB/C mice (p = 0.02). CpG-B did not significantly change IFNα in NOD or BALB/c mice but increased TNFα (p = 0.05) in both mice lines. Treatment with BL-7040 decreased TNFα and IFNα levels in NOD mice (p = 0.014). IFNα levels were decreased in NOD (p = 0.0022) but did not change in BALB/c mice treated with BL-7040. Bars are mean±SEM.

Previous research suggests that the initiation of Sjögren's Syndrome (SjS) involves production of RNA- or DNA-containing immune complexes by local apoptotic processes. These complexes induce IFNα, causing local activation of pDC and B cells in the salivary glands, production of yet more IFNα, and enhanced infiltration of T-cells and macrophages into the diseased glands [9], [24]. NOD mice, used as a model to study SjS features [25], presented reduced salivary functioning compared to BALB/c controls (p = 0.05; Fig. 2A), and their salivary glands showed higher levels of cleaved caspase-3 (p = 0.05), suggesting accelerated apoptosis (Fig. 2B).

Different CpG ODNs initiate different physiological mechanisms capable of controlling both inflammation and autoimmunity [26] as well as an immunosuppressive response [27]. To test whether BL-7040 activation of TLR9 plays an active role in controlling immune and salivary functioning and is modified in SjS, we tested the effect of TLR9-affecting ODNs on saliva secretion of NOD mice compared to BALB/c controls (Fig. 2C) [20]. Given that the endosomal maturation inhibitor hydroxychloroquine inhibits TLR9 activation and is a common treatment for SjS [27], we injected NOD and BALB/c mice with ODN2088, which dramatically increased salivation in NOD mice (p = 0.0018) (Fig. 2D).

The IFNα-activating CpG-A triggered a decline in salivary functioning in NOD mice compared to saline (p = 0.02), in agreement with reports that IFNα excess is associated with attenuated saliva secretion [10]. As expected, CpG-A-treated BALB/c and NOD mice also displayed increased levels of IFNα (p = 0.0001and p = 1.5*10^−7^respectively) and TNFα (p = 0.05 and p = 0.04, respectively). In comparison, NOD mice treated with CpG-B (5 µg/kg) [28] displayed significantly increased saliva secretion (p = 0.003), with unchanged IFNα and elevated TNFα levels (p = 0.05). In contrast, BL-7040 (5 µg/Kg), while markedly enhanceing saliva secretion in NOD (p = 0.001), caused no elevation in TNFα or IFNα (Fig. 2D–F). These results suggest an alternative TLR9 pathway activation which is particularly robust under autoimmune conditions.

### BL-7040 activates the alternative TLR9 pathway

**Figure 3:**
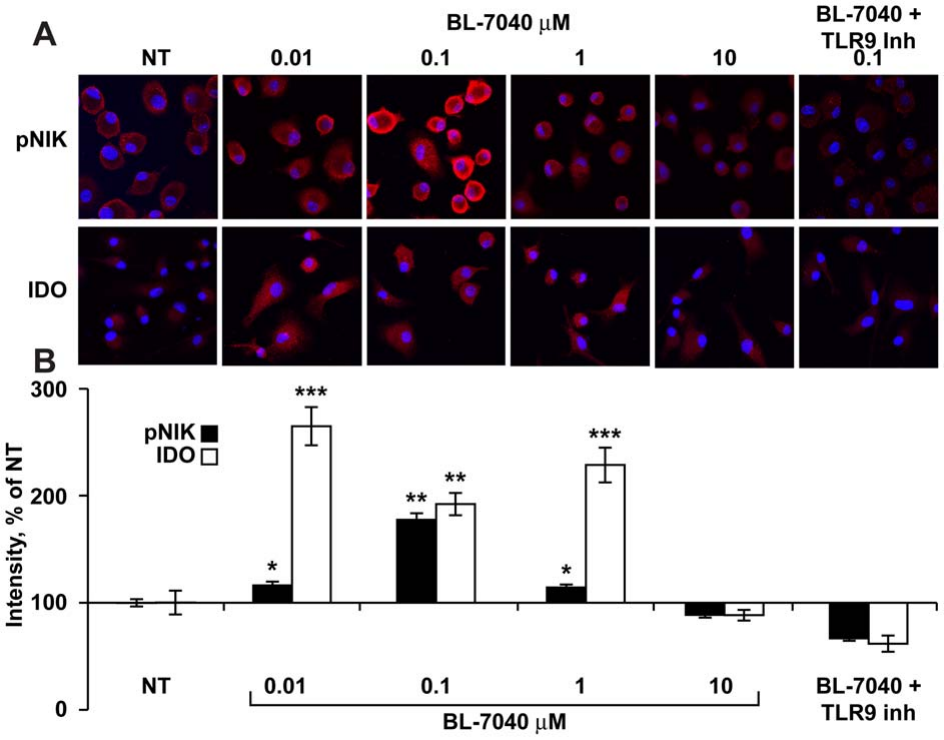
Low dose BL-7040 activates the alternative TLR9 pathway in mouse mononuclear cells. A. Immunocytochemistry for pNIK in bone marrow macrophages from C57BL mice treated for 1 hr with BL-7040 (0–10 µM), with or without TLR9 inhibitor (0.1 µM). Immunocytochemistry for IDO in bone marrow-derived dendritic cells treated with BL-7040 (0–10 µM) with or without a TLR9 inhibitor. The presented microphotographs are representative examples from 5 different samples in 3 biological repeats. Cells grown on glass slides were dried, cover-slipped, and analysed was with a 40×/1.3 oil immersion objective using a FV-1000 confocal attachment (Olympus, Japan) to an IX81 inverted microscope. Excitation wavelengths for Cy2 and Cy3 were 488 and 543 nm, respectively. Emissions were collected using the 505–525 nm and 560–620 nm filters, respectively. 50 cells were measured in each image. 3–7 images were analyzed from each treatment group. B. Quantification of immunohistochemistry for both pNIK and IDO (0.1 µM). For quantification of pNIK and IDO immuno-staining, at least three different preparations were sampled from each treatment. Using an ANALYSIS image analysis software, the labeled cells were encircled and the percent of immunostained area was calculated. P values represent non-treated vs BL-7040 treated cultures. * P<0.05, ** P<0.001, *** P<0.0005. Bars are mean±SEM.

Canonical NFκB activation induces pro-inflammatory mediators (e.g. TNFα, NO) downstream of TLR9, whereas alternative signaling involves (among others) phosphorylation of NIK [29] and may account for the apparent ability of TLR9 activation to restore homeostasis by inducing the production of proteins such as IDO. Immunocytochemistry for phosphorylated NIK (pNIK) and IDO was performed on mononuclear cells from C57BL mice treated with BL-7040 alone or together with a TLR9 inhibitor. High pNIK and IDO levels were observed following treatment with BL-7040, peaking at 0.1 µM. Co-treatment with the TLR9 inhibitor completely abolished pNIK and IDO induction, reinforcing the TLR9 dependence of this effect (Fig. 3A, 3B).

**Figure 4:**
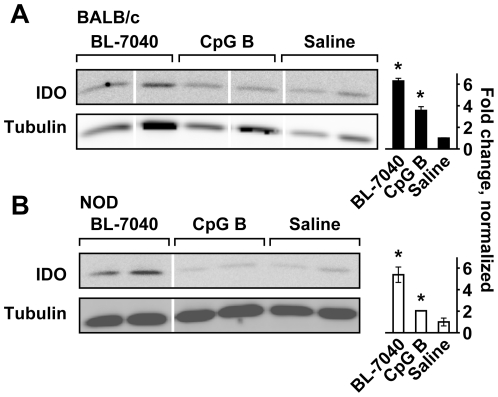
BL-7040 activates the alternative NFκB pathway in murine salivary glands. Western blots of gland extracts from BALB/c and NOD mice receiving the stated treatments are shown. Bands are taken from the same gels but from non-adjacent lanes. The western blot analysis was done on protein samples extracted from all mice tested for saliva secretion (9–12 mice per treatment), and representative gels are displayed. Analysis of the gels was done using ImageJ. A. BL-7040 and CpG-B elevated IDO (p = 0.01, 0.04) in BALB/c mice. B. BL-7040 and CpG-B increased IDO (p = 0.01, p = 0.003) in NOD mice.

These findings raised the question whether IDO and pNIK are also elevated in mouse salivary glands treated with CpG-B and BL-7040. We found that both CpG-B (p = 0.01, 0.003) and BL-7040 (p = 0.01, 0.04) elevated IDO (Fig. 4A, 4B) and pNIK levels (Figure S2A, S2B) in BALB/c (p = 0.05, 0.01) and NOD (p = 0.001, 0.05) mice. This further suggests that alternative NFκB activation is reinforced under impaired salivary conditions.

### TLR9^−/−^ mice show impaired salivary functioning

**Figure 5:**
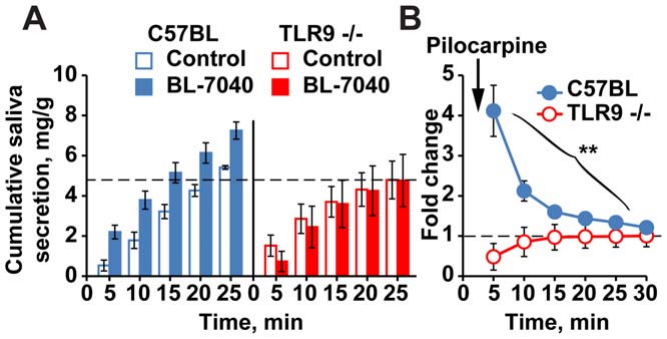
The BL-7040 salivation effect fails in TLR9^−/−^ mice. Cumulative (5A) or fold change values from time -0- of salivary activity (5B) were plotted, and the statistical significance of the difference between the measured values of wild type and TLR9-/- mice was performed using Mann-Whitney U-test, ANOVA and post-hoc analysis (Tukey). Significance was determined at the level of p<0.05. Statistical analysis was performed by SPSS 16. Normalization was confirmed by MatLab. **A**. BL-7040 increased saliva secretion in C57BL but not in TLR9^−/−^ mice (P = 0.003), which also showed lower baseline salivary secretion. Bars are mean±SEM. **B**. BL-7040 increased saliva secretion in C57BL mice but showed a decay over time, while no change was seen in TLR9^−/−^ mice. Bars are mean±SEM.

To determine if TLR9 is essential for the BL-7040 effect we treated C57BL and TLR9^−/−^ mice with BL-7040. C57BL mice showed a strong yet short-lasting salivary response. In contrast, TLR9^−/−^ mice showed consistently lower salivary activation and were totally refractory to BL-7040 treatment (Fig. 5A, 5B). Together, this demonstrates that BL-7040 responses are limited to TLR9-expressing mouse strains, albeit with strain-specific kinetics.

### Human SjS salivary glands show elevated AChE and decreased alternative markers

**Figure 6:**
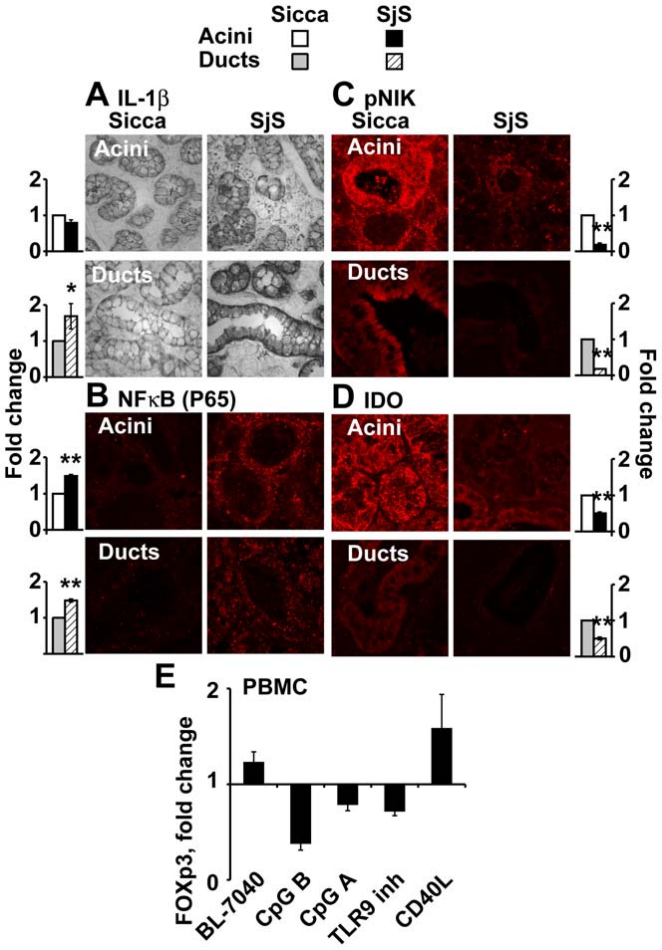
Sjogren's disease biopsies show elevated inflammatory markers and suppressed alternative NFκB markers. The presented microphotographs are representative examples from 15 different SjS patients and 12 Sicca syndrome control samples in 3 biological repeats. Biopsies stained for the specific proteins were acquired with a 40×/1.3 oil immersion objective using a FV-1000 confocal attachment (Olympus, Japan) to an IX81 inverted microscope. Excitation and emission wavelengths for Cy2 and Cy3 are as described in Figure 3A. At least 3 acini and 2 ducts were measured in each image. 12–15 images were analyzed of each treatment group. **A**. IL-1β was increased in SjS ducts (p = 0.05) but not acini compared to Sicca syndrome controls. **B**. The p65 fragment of NFκB was increased in SjS compared to Sicca syndrome controls (p = 0.0002). **C**. pNIK levels were decreased in SjS patients compared to Sicca syndrome controls (p = 0.0001). **D**. IDO levels were decreased in SjS patients compared to Sicca syndrome controls (p = 7.5*10^−12^). **E**. PBMN cells displayed elevated FOXp3 levels after activation by CD40L or BL-7040. Treatment with CpG type A, B and TLR9 inhibitor decreased FOXp3 levels.

Salivary gland biopsy sections from primary SjS patients and Sicca syndrome controls were tested for markers of TLR9 activity and cholinergic signaling. The rational for using tissue samples from SjS patients without anti-Ro/SSA and anti-La/SSB antibodies was to avoid the possibility that our observations were related to the immune response rather than to the SjS phenotype itself [30]. Although these patients were in preliminary disease stages and tested negative for anti-Ro/SSA and anti-La/SSB they displayed increased markers of the canonic TLR9 pathway, compatible with previous reports [31]: IL-1β was elevated in SjS gland ducts (p = 0.05), Fig. 6A) and NFκB (P65) was increased in both acini and ducts (p = 0.0002) (Fig. 6B). However, the alternative markers pNIK and IDO were decreased in SjS biopsies (p = 0.0001and p = 7.5*10^−12^, respectively) (Fig. 6C, 6D). Finally, in human PBMC, BL-7040 induced FOXp3 mRNA levels, known to be activated by IDO [32], as did CD40L, a known activator of Tregs. This increase was not detected with any of the other CpGs (Fig. 6E).

Last, we tested the possibility that together with infiltrating lymphoid cells and decreased saliva secretion caused by inflammation, AChE-induced suppression of cholinergic activity may “shut down” fluid secretion. Labial gland biopsies from SjS patients showed elevated AChE expression in both acini (p = 0.002) and ducts (p = 0.025) (Figure S3B). AChE-S mRNA levels remained unchanged (Figure S3D) while AChE-R mRNA levels were increased (Figure S3C). This would increase the secreted soluble monomers of the AChE-R variant, which can readily access the neuron-gland interaction sites. Thus, as in other diseases (e.g. *myasthenia gravis* [33]), AChE over-expression is accompanied by changes in alternative splicing from the common AChE-S to the normally rare AChE-R variant.

## Discussion

In our research we demonstrate that BL-7040 is a TLR9-specific ligand that can simultaneously suppress pro-inflammatory functions and shift NFκB from the pro-inflammatory canonical to the alternative pathway. We show that this activation of the NFkB alternative pathway leads to suppression of the immune system activator IFNα and increases IDO levels. We also show severe impairments of salivary functions in TLR9^−/−^ mice and demonstrate CpG-induced, TLR9-dependent restoration of this alternative pathway, which can enhance saliva secretion in malfunctioning glands of NOD mice.

IFNα is perceived as the functional bridge between pDCs and Tregs, and is linked with resistance to specific forms of immuno-pathogenesis and infection [34]. Total blockade of the IFNα pathway improves disease symptoms, as in the case of SjS patients treated with Hydroxychloroquine. This compound attenuates endosomal maturation [27]; however, since the endosome is important for many other cellular functions, its chronic systemic blockade might have both beneficial and harmful effects [35], [36]. In contrast, BL-7040, with its unique chemical protection pattern and multimeric structure, should be considered as an advantageous therapeutic entity (Figure S4).

That both inhibition (by a TLR9 inhibitor) and non canonic NFkB activation (by BL-7040) decrease AChE activity suggests that either complete TLR9 blockade or TLR9-mediated alternative activation of NFkB may suppress the downstream regulator(s) controlling AChE levels (such as microRNA-132 [37]); that both of these together have no effect suggests that the two pathways most likely use common adaptor protein(s) (e.g. MyD88) to contact the regulators.

The autonomic nervous system releases ACh continuously from the vagus, activating the M3R receptors in salivary gland cells and leading to saliva production. A finely-tuned balance between this brain-to-body signaling process and secretion of pro-inflammatory cytokines, which attenuate saliva production, is essential for proper regulation of this autonomous function. The fact that salivary gland cells isolated from SjS patients show over-expression of M3R could reflect a feedback response to disease-associated suppression of ACh release [12], [20]. This suppression could be due to enhanced ACh degradation by AChE which terminates cholinergic signaling [13]. In accordance, we show that salivary gland biopsies from SjS patients display higher levels of AChE compared to Sicca syndrome controls. Additionally, age-related increases in circulation AChE and pro-inflammatory cytokines [38] indicate parallel acceleration of salivary mal-functioning. The consistently elevated AChE in females compared to males [39] may be relevant for the gender-related incidence of SjS [1]. General impairments in cholinergic signaling and consequent enhancement of immune functions may hence facilitate SjS glandular hypofunction, disparate from the effect of anti-M3R auto-antibodies, which cause at least part of the SjS symptoms. Our current study raises the possibility of an inter-relationship between TLR9, cholinergic and immune functions as a powerful mechanism for monitoring saliva secretion.

IKKα, which is downstream to TLR9, is required for the development of self-tolerance, whereas IKKβ mediates NFκB activation in response to pro-inflammatory stimuli and is crucial for canonic pathway activation [40], [41], [42]. We show that SjS patient biopsies have an altered balance between canonic and alternative NFκB signaling. IL-1 and NFκB (P65) – canonic pathway markers – are elevated, whereas pNIK and IDO – alternative pathway markers – are reduced. TLRs, NFκB and IDO are intimately linked in the prevention of immuno-pathogenesis that involves function of Tregs [10], [43] and elevated IDO levels have been shown to activate Tregs, known to maintain tolerance to self antigens [10]. We show that the Treg-characteristic FOXp3 translational activator was increased as a result of BL-7040 treatment of human Peripheral blood mononuclear cells. This could potentiate the alternative pathway, leading to resolution of early inflammatory processes and to the onset of tolerance to self [40], [44].

In conclusion, TLR9 activation of the NFκB canonical pathway can lead to constitutive over-production of pro-inflammatory cytokines and weakened cholinergic signaling. Parallel phenomena are associated with a number of chronic inflammatory disorders, including rheumatoid arthritis and Crohn's disease [45]. Thus, interference with neuroimmune impairments, including in early stages of SjS, should be targeted to both their neuronal and immune aspects.

Although the focus of our manuscript is of a basic nature we suggest that it may have therapeutic applications. There are still many questions left unanswered regarding the clinical aspects of BL-7040, including a more detailed clinical and histolopathological assessment of the patients, analysis of AChE levels and local IFNα production, and the association of non-canonical NFkB markers with different stages of Sjogren's syndrome. Further studies will hence be required to determine if BL-7040 could be beneficial for these syndromes as well as for neurodegenerative diseases in which neuroinflammation is involved, such as Alzheimer's or Parkinson's diseases [46], [47].

## Materials and Methods

### Human labial gland samples

Labial glands were collected from the lower lip of 15 SjS patients (with pathological diagnosis, without ribonuclear antibodies) and 12 Sicca syndrome patients under local anaesthesia at the Queen Elizabeth Hospital (Woodville South, Australia). SjS patients were defined according to the revised 2002 American European Classification criteria (Table S1). The clinical definition of SjS was largely based on the salivary malfunctioning criterion as assessed by expert physicians. The study was approved by the local Institutional Review Board (Application number 2008124, see description of patients in Table S1).

### Mice

Female Non-Obese Diabetic (NOD) mice and control BALB/c and C57BL mice (approximately 5 months old) were obtained from Harlan (Rehovot, Israel). While NOD mice also show characteristic features of diabetes, these progress at a later age than that used for our current study [25], and there were no insulin injections involved which could potentially influence the TLR modulations. TLR9^−/−^ mice were gratefully received from Dr. Eithan Galun, (HU-HMS Jerusalem). 9–12 mice per experimental group were employed. Salivation experiments were repeated 3 times, with 3–4 mice per treatment. All experiments were carried out according to the Animal Care and Use Committee of The Hebrew University, Jerusalem (approval # NS-04-21).

### Mouse submandibular gland samples

Mice were sacrificed by cervical dislocation and their submandibular salivary glands were removed and immediately homogenized in 200 µl per gland of a high-salt/detergent buffer (0.01 M Tris, 1 M NaCl, 1% Triton X-100, 1 mM EGTA, pH 7.4) [37].

### Cells

HEK-293 and the murine macrophage-derived cell line RAW 264.7 were used. A human salivary gland (HSG) cell line was gratefully received from Dr. A. Palmon (HU-HMS, Jerusalem). Human and mouse primary mononuclear cells were prepared as described previously [37].

### TLR screening

TLR activators were tested on recombinant HEK-293 cell lines stably expressing a given functional TLR protein as well as a luciferase reporter gene driven by the NFκB promoter. HEK293 cells express no TLRs, which makes it an excellent cell line for over-expression of exogenous TLRs. The exogenous expression of the noted TLRs was validated in each cell line separately by specific qPCR assays (data not shown). TLR activation results are given as optical density values (OD). For positive controls, each HEK293-TLR cell line was induced with a ligand specific to the expressed transgenic TLR. Positive control ligands were: TLR2; PAM2 (100 ng/ml), TLR3; Poly I:C (100 ng/ml), TLR4; LPS K12 (100 ng/ml), TLR5; Flagellin (1 µg/ml), TLR7; R848 (10 µg/ml), TLR8; R848 (10 µg/ml), TLR9; ODN 2006 (CpG-B) (10 µg/ml). 20 µl of each ligand was used to stimulate cell lines in 200 µl reaction volume. BL-7040 concentration for induction was 100 µM. The negative control was a HEK-293 cell line expressing the reporter gene alone. For each cell line the reporter signal under non-induced conditions was taken as the baseline value.

### Immunocytochemistry, in situ hybridization and cholinesterase catalytic activities

Polyclonal antibodies against IL-1β (R&D, Abingdon, UK, 1∶20 dilution); AChE N19 (Santa Cruz Biotechnology Inc., Santa Cruz, CA, USA, 1∶20), pNIK (Santa Cruz, 1∶50), FOXP3 (eBioscience, San Diego, CA, USA, 1∶50) and IDO (Millipore, Billerica, MA, USA, 1∶50), were used as described [48]. In-situ hybridization and cholinesterase activity measurements were carried out as described previously [46].

### Oligonucleotides

BL-7040 was obtained from Avecia (Cambridge, UK) and ODN 1826, 1585, 2088, 2006, 2216 and TTAAG from Invivogen (San Diego, California, USA). All were endotoxin-free. Table S2 shows the sequences and chemical protection modes of the ODNs.

### Mouse saliva secretion

Mice (n = 11 per group) were intraperitoneally administered with various ODNs (Table S2) or saline for four consecutive days. On the fourth day, two hours after ODN injection, mice were anesthetized with Ketamine-Xylazine (100 mg/Kg and 10 mg/Kg, i.p) and injected with 15 mg/kg pilocarpine to induce salivation. Mice were placed on an inclined surface with their heads facing downward and a pre-weighed piece of Whatmann paper was placed in their mouth. Papers were changed and weighed every 5 min (last change at 30 min post-pilocarpine injection).

### Western Blots

Salivary gland homogenates were separated by standard SDS-PAGE as previously described [48]. Proteins were visualized using antibodies to pNIK (1∶500), α-Tubulin (1∶1000) (sc-12957 and sc-32293, Santa Cruz) and IDO (1∶500) (AB9900, Millipore, Billerica, MA) followed by peroxidase-conjugated antibodies (1∶10,000, Jackson Laboratories, West Grove, PA) and enhanced chemiluminescence (ECL) (EZ-ECL, Biological Industries, Beit-Haemek, Israel).

### Inflammatory biomarkers

To determine NO production in macrophage cultures the stable breakdown product nitrite was measured using the Griess method [25]. 100 µl of each sample +100 µl of Griess reagent (1% sulfanylamide/0.1% naphthylethylenediamine dihydrochloride/2.5% H_3_PO_4_) were incubated for 10–20 min at room temp. and absorbance (546 nm) was determined using a microplate reader (Genios Pro, Tecan, Maennedorf, Switzerland). IFN-α and TNF-α were measured by ELISA according to manufacturer's procedures (PBL interferon source, NJ and eBioscience, Hatfield, UK).

### Confocal Microscopy

Cy2 and Cy3 fluorescence in tissue sections were determined using an FV-1000 confocal attachment (Olympus, Japan) to an IX81 inverted microscope. Excitation wavelengths for Cy2 and Cy3 were 488 and 543 nm, respectively. Emissions were collected using the 505–525 nm and 560–620 nm filters, respectively. At least 10 acini and 6 ducts per slide were analyzed.

### Statistical analysis

Statistical analysis was performed by SPSS using unpaired Student's t-test, Mann-Whitney U-test, ANOVA and post-hoc analysis (Tukey). Significance was determined at the level of p<0.05. Normalization was confirmed by MatLab.

## Supporting Information

Figure S1 **BL-7040 characterization.** A. Heating of BL-7040 (95°C for 10 minutes) did not abolish induced nitrite production by RAW 264.7 cells. B. Mass spectrometry of BL-7040 reveals spontaneous dimerization in PBS. C. Nitrite production in RAW 264.7 cells following exposure to BL-7040, phosphorothioated BL-7040 (PS), BL-7040 with sequence alterations in ends or center region and negative control oligonucleotide. Bars are mean±SEM.
(TIF)

Figure S2 **Western blots of gland extracts from treated mice. Elevation of pNIK suggests homeostatic TLR9 effects on salivary glands.** **A**. BL-7040 and CpG-B ODN1826 elevated pNIK (p = 0.01, 0.05) in BALB/c mice. **B**. BL-7040 and CpG-B ODN1826 increased pNIK (p = 0.001, p = 0.05) in NOD mice.
(TIF)

Figure S3 **Sjogren's syndrome patient biopsies show a shift in AChE splicing.** **A**. AChE pre-mRNA can undergo alternative splicing giving rise to the predominant AChE-S and the disease-inducible AChE-R splice variants. **B**. AChE protein levels increase in both acini (p = 0.04) and ducts (p = 0.02) in biopsies of SjS patients compared to Sicca syndrome controls **C**. AChE-R mRNA (p = 0.002, p = 0.025) but not AChE-S mRNA was increased in both SjS acini and ducts.
(TIF)

Figure S4 **Summary figure.** The scheme shows three alternative routes for downstream activation of TLR9-mediated signals. Type-A CpG ODNs activate the IRF7 pathway increasing IFNα levels (shown in blue). Type-B CpG ODNs induce the canonic NFkB pathway (shown in red), and BL-7040 can activate the homeostatic NFκB pathway (shown in black) similarly to CD40L, elevating IDO levels and possibly Treg activation.
(TIF)

Table S1 **List of biopsies from SjS patients.**
(DOC)

Table S2 **CpG sequences.**
(DOC)
