# Supplementary figures and images for: Activation of the Alternative NFκB Pathway Improves Disease Symptoms in a Model of Sjogren's Syndrome
Source: PLoS One. 2011 Dec 9;6(12):e28727. doi: 10.1371/journal.pone.0028727 (PMC3235165; doi:10.1371/journal.pone.0028727)

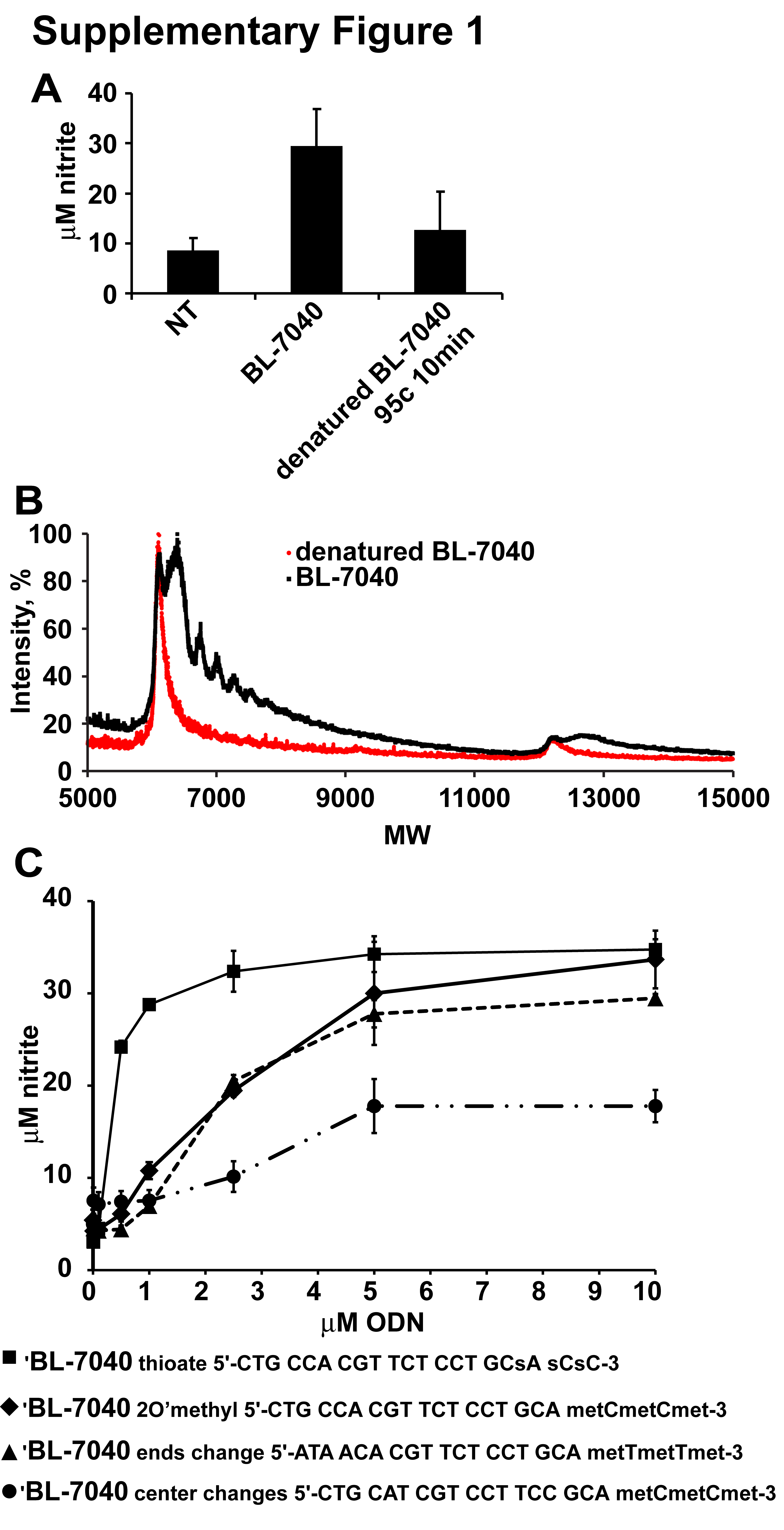

Supplement: Figure S1 — BL-7040 characterization. A. Heating of BL-7040 (95°C for 10 minutes) did not abolish induced nitrite production by RAW 264.7 cells. B. Mass spectrometry of BL-7040 reveals spontaneous dimerization in PBS. C. Nitrite production in RAW 264.7 cells following exposure to BL-7040, phosphorothioated BL-7040 (PS), BL-7040 with sequence alterations in ends or center region and negative control oligonucleotide. Bars are mean±SEM. (TIF) [file pone.0028727.s001.tif]

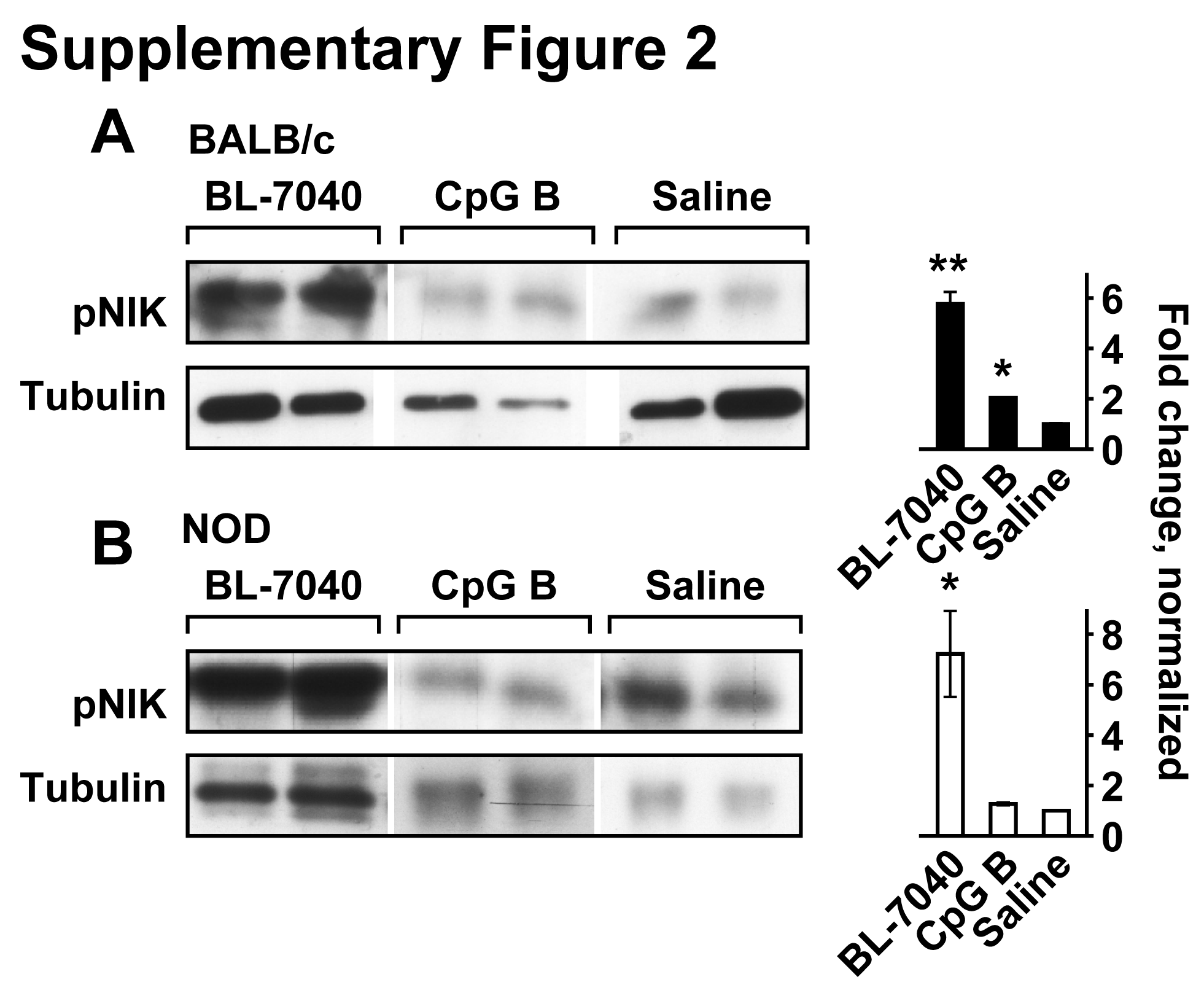

Supplement: Figure S2 — Western blots of gland extracts from treated mice. Elevation of pNIK suggests homeostatic TLR9 effects on salivary glands. A. BL-7040 and CpG-B ODN1826 elevated pNIK (p = 0.01, 0.05) in BALB/c mice. B. BL-7040 and CpG-B ODN1826 increased pNIK (p = 0.001, p = 0.05) in NOD mice. (TIF) [file pone.0028727.s002.tif]

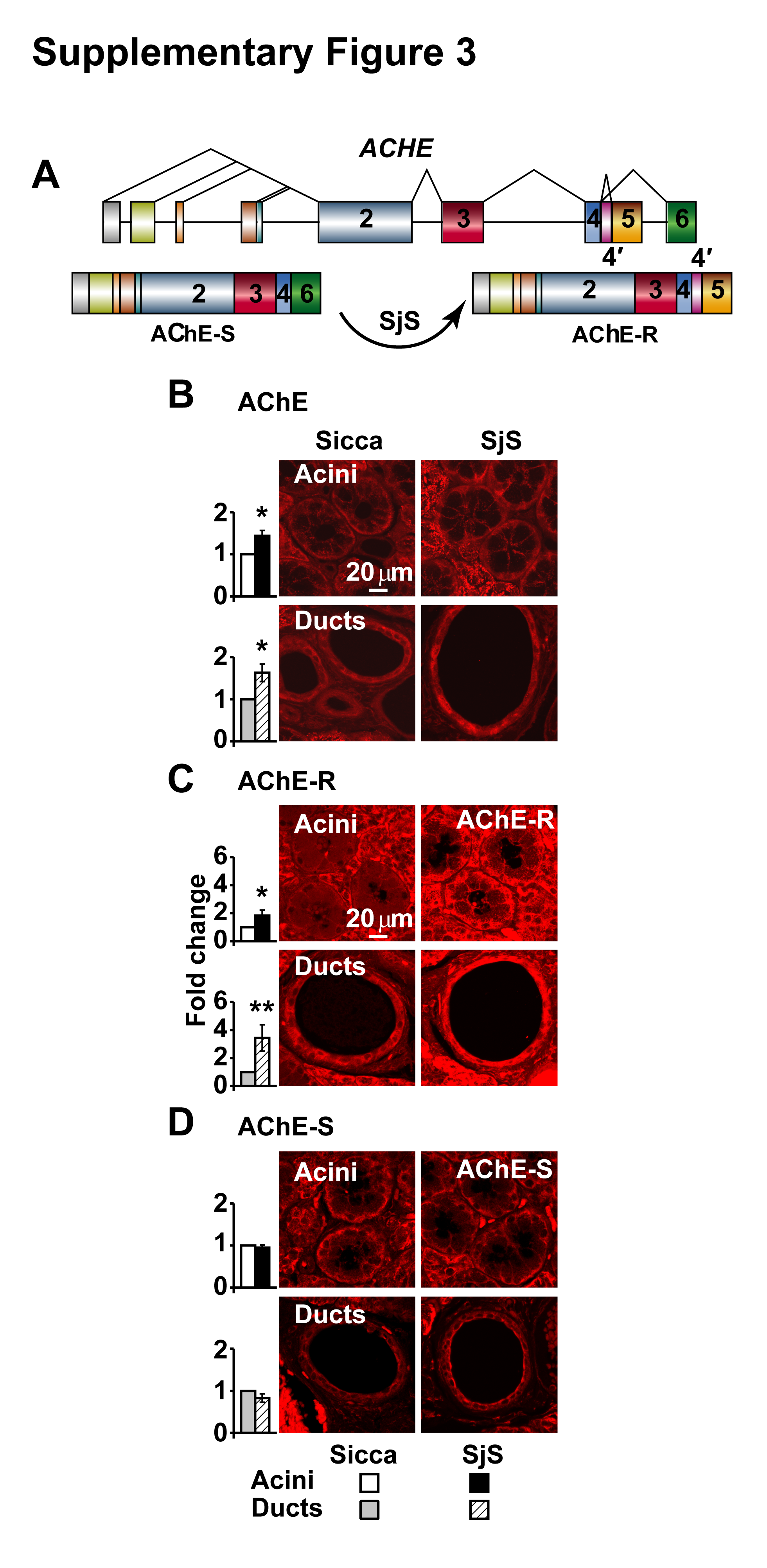

Supplement: Figure S3 — Sjogren's syndrome patient biopsies show a shift in AChE splicing. A. AChE pre-mRNA can undergo alternative splicing giving rise to the predominant AChE-S and the disease-inducible AChE-R splice variants. B. AChE protein levels increase in both acini (p = 0.04) and ducts (p = 0.02) in biopsies of SjS patients compared to Sicca syndrome controls C. AChE-R mRNA (p = 0.002, p = 0.025) but not AChE-S mRNA was increased in both SjS acini and ducts. (TIF) [file pone.0028727.s003.tif]

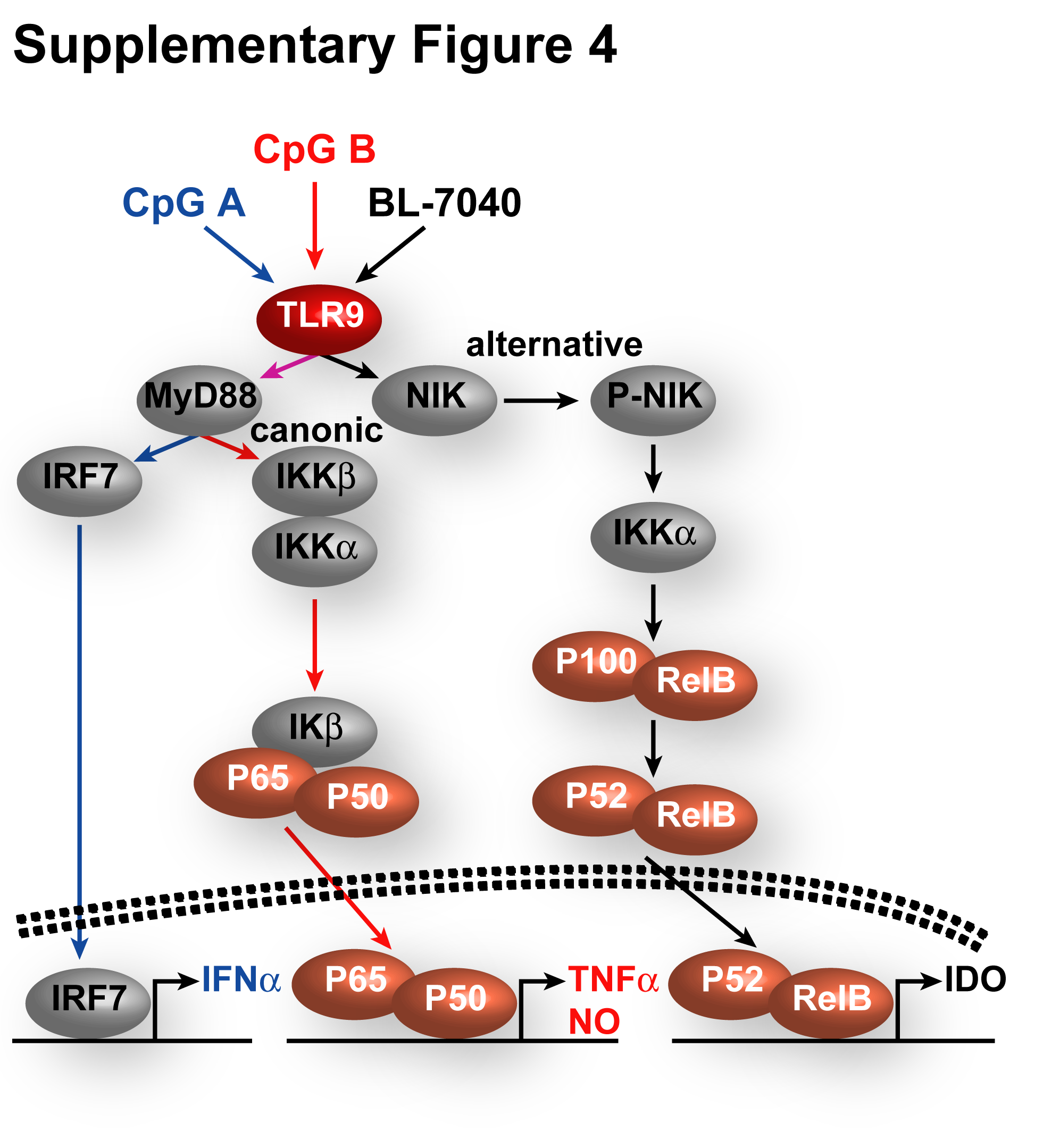

Supplement: Figure S4 — Summary figure. The scheme shows three alternative routes for downstream activation of TLR9-mediated signals. Type-A CpG ODNs activate the IRF7 pathway increasing IFNα levels (shown in blue). Type-B CpG ODNs induce the canonic NFkB pathway (shown in red), and BL-7040 can activate the homeostatic NFκB pathway (shown in black) similarly to CD40L, elevating IDO levels and possibly Treg activation. (TIF) [file pone.0028727.s004.tif]
