# Supplementary material for: Activation of the Alternative NFκB Pathway Improves Disease Symptoms in a Model of Sjogren's Syndrome
Source: PLoS One. 2011 Dec 9;6(12):e28727. doi: 10.1371/journal.pone.0028727 (PMC3235165; doi:10.1371/journal.pone.0028727)
Supplement: Table S1 — List of biopsies from SjS patients. (DOC) [file pone.0028727.s005.doc]

**Table S1: List of biopsies from SjS patients.**

| Clinical diagnosis | Pathological diagnosis * | Slide ID | SS database# |
| --- | --- | --- | --- |
| Definite primary SjS | 3.7 | 3538645/00 | 567 |
| Definite primary SjS | 2 | 3540495/99 | 281 |
| Definite primary SjS | >1 | 3542597/99 | 267 |
| Definite primary SjS | 2 | 3534813/00 | 287 |
| Definite primary SjS | >2 | 03-501138 | 596 |
| Definite primary SjS | 2-3 | 00/s07327 | 539 |
| Definite primary SjS | 2.5 | 98/s08878 | 151 |
| Definite primary SjS | 3 | 00/s02036 | 251 |
| Definite primary SjS | >6 | 99/s02634 | 158 |
| Definite primary SjS | >1 | 00/s02075 | 253 |
| Definite primary SjS | 2 | 98/s08731 | 148 |
| Definite primary SjS | 2 | 11373 | 79 |
| Definite primary SjS | Negative | 18216/99 | 231 |
| Definite primary SjS | Negative | 1085591 | 190 |
| Definite primary SjS | Negative | V15598-99 | 194 |
| Sicca syndrome | Normal | 8530124/01 | 576 |
| Sicca syndrome | Normal | 872078/02 | 636 |
| Sicca syndrome | Normal | 02-526311 | 677 |
| Sicca syndrome | Normal | 98/S08587 | 147 |
| Sicca syndrome | Non-specific | 8534437/01 | 599 |
| Sicca syndrome | Non-specific | 8539484/01 | 631 |
| Sicca syndrome | Non-specific | 3539793/00 | 575 |
| Sicca syndrome | Non-specific | 02-526599 | 714 |
| Sicca syndrome | Normal | 02-526198 | 711 |
| Sicca syndrome | Normal | 02-501579 | 630 |
| Sicca syndrome | Normal | 01-524634 | 624 |
| Sicca syndrome | Non-specific | 98/S08877 | 150 |

Pathological diagnosis characterized as Focal sialadenitis, focus score per 4mm^2^ of tissue.

Clinical diagnosis was defined according to the revised 2002 American European Classification criteria (Vitali C, Bombardieri S, Jonsson R, Moutsopoulos HM, Alexander EL, Carsons SE,Daniels TE, Fox PC, Fox RI, Kassan SS, Pillemer SR, Talal N, Weisman MH; European Study Group on Classification Criteria for Sjögren's Syndrome. Classification criteria for Sjögren's syndrome: a revised version of the European criteria proposed by the American-European Consensus Group. Ann Rheum Dis. 2002 Jun;61(6):554-8).
