# Supplementary material for: Activation of the Alternative NFκB Pathway Improves Disease Symptoms in a Model of Sjogren's Syndrome
Source: PLoS One. 2011 Dec 9;6(12):e28727. doi: 10.1371/journal.pone.0028727 (PMC3235165; doi:10.1371/journal.pone.0028727)
Supplement: Table S2 — CpG sequences. (DOC) [file pone.0028727.s006.doc]

Table S2: CpG sequences.

| ODN | Sequence | Dose | Chemical Protection |
| --- | --- | --- | --- |
| ODN1826 | 5’tcc atg acg ttc ctg acg tt -3’ | 500 µg/Kg,  5 µg/Kg | Fully phophoro-thiated |
| ODN1585 | 5’ggg gtc aac gtt gag ggg gg 3’ | 500 µg/Kg | Fully phophoro-thiated |
| ODN2088 | 5’tcc tgg cgg gga agt -3 | 500 µg/Kg | Fully phophoro-thiated |
| ODN2216 | 5’ggg gga cga tcg tcg ggg gg -3’ | 1 µM | Fully phophoro-thiated |
| ODN2006 | 5’tcg tcg ttt tgt cgt ttt gtc gtt -3 | 0.1 µM,  1 µM | Fully phophoro-thiated |
| ODN TTAGGG | 5’ttt agg gtt agg gtt agg gtt agg g -3’ | 1 µM | Fully phophoro-thiated |
| BL-7040 | 5’-ctg cca cgt tct cct gca *c*c*-3’  *2 O’-methyl | 500 µg/Kg,  5 µg/Kg  0.1 µM,  1 µM | 3’-2-O-methyl protected |
